# Spatial analysis of transcript and protein levels in skeletal muscle

**DOI:** 10.1016/j.xpro.2024.103378

**Published:** 2024-10-09

**Authors:** Paola Pisterzi, Clara Martinez Mir, Ouafa Dahri, Isabel de Poorter, Sandra Batlles Parera, Milica Dostanić, Massimo Mastrangeli, Christine Mummery, Niels Geijsen, Fanny Sage

**Affiliations:** 1 Department of Anatomy and Embryology, Leiden University Medical Center, 2333 Leiden, the Netherlands; 2 The Novo Nordisk Foundation Center for Stem Cell Medicine (reNEW), Leiden Node, the Netherlands; 3 Microelectronics Department, Delft University of Technology, 2628 CD Delft, the Netherlands

**Keywords:** Microscopy, Gene Expression, *In Situ* Hybridization, Tissue Engineering

## Abstract

Skeletal muscle spatial analyses have revealed unexpected regionalized gene expression patterns challenging the understanding of muscle as a homogeneous tissue. Here, we present a protocol for the spatial analysis of transcript and protein levels in murine skeletal muscle. We describe steps for tibialis anterior dissection, formaldehyde fixation, tissue chopper cutting, and hybridization chain reaction (HCR) detection and amplification. We then detail procedures for immunostaining, tissue clearing, and imaging. This protocol is easily adaptable to other tissues.

## Highlights

• Detailed protocol for spatial analysis of transcripts and proteins in murine TA muscle
• Steps for obtaining optimal thick sections of fixed TA muscle
• Guide for hybridization chain reaction (HCR) detection and amplification
• Steps for immunostaining, clearing, and confocal imaging of thick muscle sections

## Abstract (editor highlights)

Publisher’s note: Undertaking any experimental protocol requires adherence to local institutional guidelines for laboratory safety and ethics.

## Graphical abstract

**Figure:**
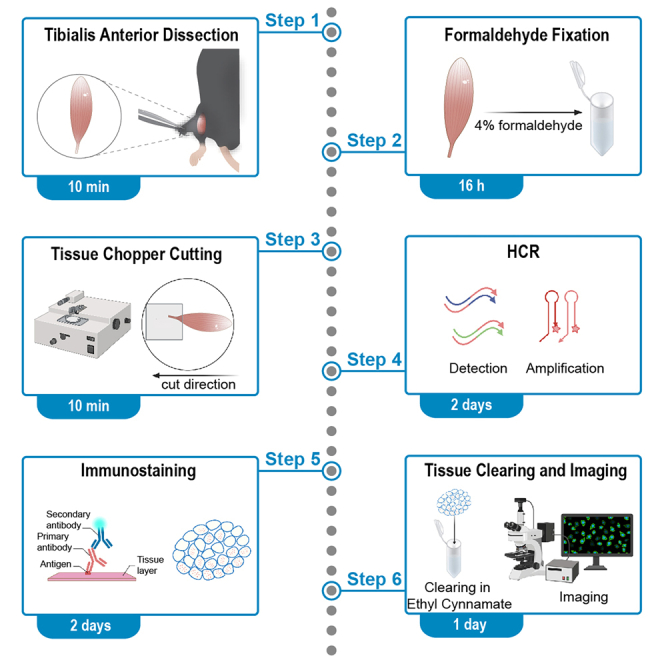

## Before you begin

This protocol describes steps specifically performed using thick sections of the mouse tibialis anterior (TA) muscle, as it is a readily accessible tissue frequently used for *in vivo* research. However, these steps are suitable for other muscles (e.g., extensor digitorum longus [EDL], soleus and gastrocnemius) as well as for *in vitro* tissue engineered muscle (TEM), as shown in the last part of this protocol.

### Institutional permissions

TA muscles were obtained from 8-month-old Tg:Pax7-nGFP male mice^1^ (kindly provided by Dr. S. Tajbakhsh, Pasteur Institute Paris, France). Experiments were performed in accordance with national regulations and approved by the Animal Experiment Committee of the Leiden University Medical Center.

Note that this protocol requires the collection of murine tissues, therefore before performing the following procedures, permission should be acquired from the relevant institutions.

### Probe design and validation

**Timing: 3 weeks**

The following protocol requires the design and validation of hybridization chain reaction (HCR) probes. HCR requires multiple pairs of DNA probes and fluorescently labeled hairpins (h1 and h2). Each probe pair hybridizes to the RNA target and contains half of an initiator sequence. When a probe pair binds to the RNA target, the full initiator sequence is exposed and recognized by hairpin h1, causing it to unfold and reveal a domain for hairpin h2. Hairpin h2 then unfolds, exposing an initiator-like sequence. This triggers an enzyme-free chain reaction, where both hairpins bind to each other, amplifying the fluorescent signal and enabling visualization of RNA targets in the sample.

To execute this protocol, we have requested probe design from Molecular Instruments, however probe design and synthesis could be performed independently.^2^

1. Request probe design from Molecular Instruments, Inc. (https://www.molecularinstruments.com/) and order probe sets, amplifiers, and buffers.
  a. To obtain specific probes for the RNA target sequence, provide the NCBI accession number of the gene of interest.
    i. Select a probe size of 20 pairs to ensure optimal detection. If not possible, a smaller probe size can still provide good detection levels.
    ii. Choose matching amplifier and probe set. Use different amplifiers when imaging multiple targets in the same sample, for example use B1 for probe set 1, B2 for probe set 2, and so on.
    iii. Avoid spectral overlap by carefully choosing the fluorophore of each amplifier if you are considering using multiple probes in a single assay or combining *in situ* HCR with immunostaining.
2. Validate probes.
  a. Test probe sets using concentrations ranging from 4 to 16 nM, according to manufacturer instructions.

***Note:*** Evaluate probe specificity by testing the probe set on tissues that do not express the target. Expression pattern can be predicted according to cell types (e.g., Titin [*Ttn*] expression is expected in myonuclei, but not in satellite cells).

### Experimental design

Thick sections obtained from murine TA muscle are stained to detect *Ttn* expression by HCR and immunolabeled with antibodies against GFP to identify Pax7-nGFP^+^ satellite cells, laminin to mark the basal lamina, and DAPI to highlight the nuclei.

As negative control, one section is incubated with hairpin h1 and h2, secondary antibodies and DAPI.

***Note:*** Additional controls could be considered to account for tissue autofluorescence, these include a) an unstained sample, b) isotype controls matching the primary antibodies used, c) a sample incubated with primary antibodies only, d) a sample incubated with the *Ttn* probe set only. Furthermore, samples labeled with individual fluorophores (single staining controls) could be used to determine the extent of spectral overlap between the channels. A control for probe specificity could also be included, by testing the probe set on a tissue which does not express the target.

**Table:** 

Probe set	Hairpin h1 and h2	Fluorophore
*Ttn*	B2	647

**Antibody**		

αGFP	–	488
αLaminin	–	594

**Nuclear staining**		

DAPI	–	405

## Key resources table

**Table:** 

REAGENT or RESOURCE	SOURCE	IDENTIFIER
**Antibodies**		

Anti-laminin-2 (α-2 chain) antibody, rat monoclonal (dilution 1:200)	Sigma	L0663-.1ML
Rb pAb to GFP (dilution 1:200)	Abcam	Ab6556
Anti-myosin 4, eBioscience (dilution 1:50)	Invitrogen	14-6503-82
Alexa Fluor 488 goat anti-mouse IgG (H + L) (dilution 1:500)	Life Technologies	A11001
Alexa Fluor 594 goat anti-rat IgG (H + L) (dilution 1:500)	Life Technologies	A11007
Alexa Fluor 488 goat anti-rabbit IgG (H + L) (dilution 1:500)	Life Technologies	A11008

**Chemicals, peptides, and recombinant proteins**		

DAPI	Invitrogen	D1306
Proteinase K	QIAGEN	19133
HCR probe hybridization buffer	Molecular Instruments	N/A
HCR probe wash buffer	Molecular Instruments	N/A
HCR amplification buffer	Molecular Instruments	N/A
Goat serum	Gibco	16210064
Skim milk powder	Millipore	70166-500G
16% formaldehyde solution (w/v), methanol-free	Thermo Scientific	28908
PBS (commercial, RNase-free)	Gibco	14190-094
Bovine serum albumin	Sigma-Aldrich	A3059-50G
Tween 20	Sigma	P1379-100ML
Ethyl cinnamate	Sigma-Aldrich	112372-100G
Triton X-100	Sigma-Aldrich	X100-100ML
Methyl alcohol, anhydrous	Macron Fine Chemicals	3014-25
Glycerol, 99+%	Thermo Scientific	A16205.0F
Ambition nuclease-free water	Invitrogen	AM9932

**Experimental models: Cell lines**		

Myoblasts isolated from hindlimbs of wild-type C57BL/6 mice	N/A	N/A

**Experimental models: Organisms/strains**		

Tg:Pax7-nGFP mice	Sambasivan et al.^1^	MGI:5308730

**Oligonucleotides**		

Mouse *Ttn* probe set B2, set size: 20	Molecular Instruments	N/A
B2-647 amplifier	Molecular Instruments	N/A
Corning Matrigel growth factor reduced (GFR) basement membrane matrix	Merck	CLS356231
Gibco collagen I, rat tail	Thermo Fisher Scientific	A1048301
Horse serum	Gibco	26050070
FBS	Serana	S-FBS-CO-015
DMEM	Gibco	41966052
Ham F10	Invitrogen	31550023
bFGF	PeproTech	450-33

**Software and algorithms**		

Leica LAS X	Leica Microsystems	RRID:SCR_013673
Adobe Photoshop	Adobe Systems Inc.	RRID:SCR_014199
Adobe Illustrator	Adobe Systems Inc.	RRID:SCR_010279
Fiji	http://fiji.sc	RRID:SCR_002285

**Other**		

SecureSeal imaging spacer 8 well, diameter × thickness 9 × 0.12 mm depth 25 × 52 mm OD QTY 100/pack	Grace Bio-Labs	GBL654008-100EA
SuperFrost Plus microscope slides 72 stück/pieces 25 × 75 × 1.0 mm	VWR	631-0108
Microscope cover glasses 100 st./pcs. 24 × 60 mm	Marienfeld Superior	0101242
Costar 48-well clear TC-treated multiple well plates, individually wrapped, sterile	Corning	3548
SAPPHIRE 8-cap strip, PP, violet, domed, for 6732XX, 125 pieces/bag	Greiner Bio-One	373277
SAPPHIRE PCR 8-tube strips, 0.2 mL, PP, blue, without cap, 125 pieces/box	Greiner Bio-One	673274
RNaseZAP	Invitrogen	AM9780
1.5 mL screw neck vial	VWR	548-0385A
Screw cap, ND10, 10–425, closed top, PP, black, liner: red-orange natural rubber/transparent TEF, 1.3 mm, 60° shore A	VWR	548-3256A
SafeSeal micro tube 2 mL	Sarstedt	72.695.500
Cole-Parmer Stuart 3D gyrating rocker	Fisher Scientific	10758995
McIlwain tissue chopper	Cavey Laboratory Engineering Co. Ltd.	N/A
ThermoMixer C	Eppendorf	5382000015
SP8 laser confocal microscope	Leica Microsystems	N/A
Leica microscope objective HCX PL APO 63×/1.20 W CORR	Leica Microsystems	N/A

## Materials and equipment

**Table:** PBS-MT

Reagent	Final concentration	Amount
Skim milk	1%	2.5 g
TritonX-100	0.4%	1 mL
BSA	0.2%	0.5 mL
Goat serum	0.1%	0.250 mL
PBS	N/A	Up to 250 mL
**Total**	**N/A**	**250 mL**

Store at 4°C, maximum 2 weeks.

**Table:** Proteinase K solution

Reagent	Final concentration	Amount
Proteinase K (20 mg/mL)	10 μg/mL	0.5 μL
Tris-HCl pH 8.0 (1 M)	30 mM	30 μL
ddH_2_O	–	969.5 μL
**Total**	**N/A**	**1 mL**

Prepare fresh before use.

**Table:** 5× SSC-T buffer

Reagent	Final concentration	Amount
SSC (20×)	5×	12.5 mL
Tween-20	0.1%	50 μL
ddH_2_O	–	37.45 mL
**Total**	**N/A**	**50 mL**

Store at 19°C–22°C.

• Formaldehyde solution 4%: to make a 10 mL solution dilute 2.5 mL of 16% formaldehyde solution in 7.5 mL of ddH_2_O.

Diluted formaldehyde solution can be stored at −20°C for up to 6 months.

• PBS-Tween: 0.05% Tween-20 in 1× PBS; add 250 μL of Tween-20 to 500 mL ddH_2_O.

Store at 19°C–22°C , for up to 6 months.

• PBS-Triton: 0.4% Triton X-100 in 1× PBS; dilute 200 μL of TritonX-100 in 49.8 mL of PBS.

Store at 19°C–22°C, for up to 6 months.

**CRITICAL:** Formaldehyde solution is a hazardous reagent, it should be prepared and handled in a fume hood.

This protocol requires the use of several equipment, including a McIlwain Tissue Chopper, a ThermoMixer C, and an SP8 confocal microscope, reported in the key resources table. However, steps can be performed using comparable tools available in the lab.

## Step-by-step method details

### TA dissection

**Timing: 10 min**

The following steps describe the dissection of the TA muscle (Methods video S1).

**Figure 1:**
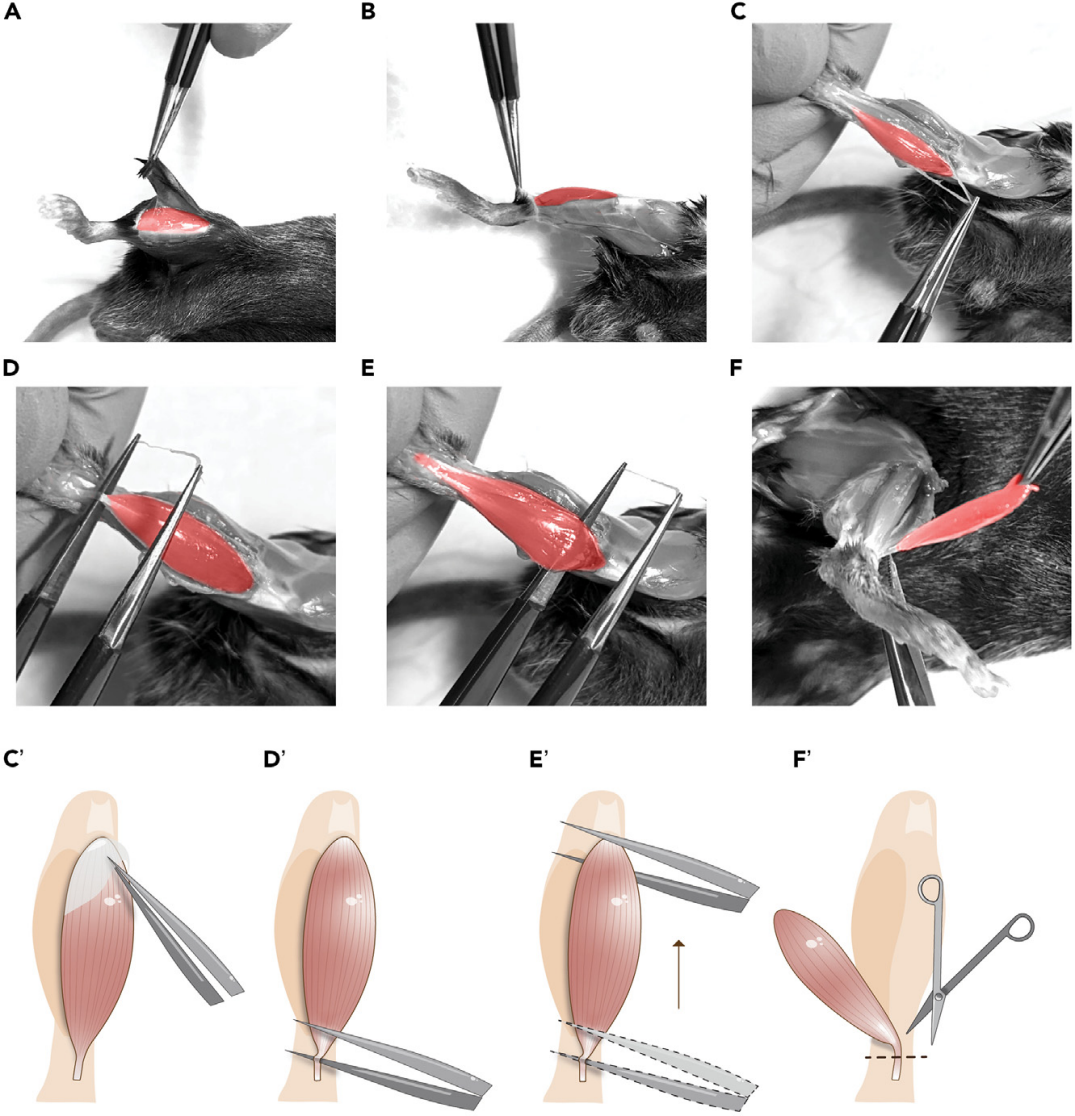
TA dissection (A–F) Removal of the skin and fascia to expose the muscle (upper panels). Insertion of forceps under the proximal TA tendon, sliding forceps towards the knee to release the muscle, and cutting the proximal tendon (lower panels). TA is highlighted in red. (C’–F’) Graphical representation of TA dissection.

1. Wipe all dissection tools with RNaseZap.
2. Euthanize the mouse according to authorized institutional guidelines.
3. Spray the hindlegs with 70% EtOH.
4. Make a small incision and remove the skin, exposing the tendons on the paw (Figures 1A and 1B).
5. Using forceps, gently remove the fascia protecting the muscle (Figures 1C and 1C’).
6. Place the forceps under the TA tendon (Figures 1D and 1D’).
  a. Slide them up towards the knee to free the muscle from the underlying tibia (Figures 1E and 1E’).
  b. Once the forceps slide easily, release the muscle from the knee joint with a firm movement (Methods video S1).

***Note:*** If when sliding the forceps under the TA there is resistance, some fascia might still be present, try to remove it before attempting to release the muscle, or the tissue will be damaged.

***Note:*** To minimize tissue damage, dissection near the knee may be performed with microscissors. In this case, first cut the distal TA tendon, then gently lift the muscle and dissect it near the knee.

7. Hold the muscle with forceps and cut the tendon from the ankle (Figures 1F and 1F’).

***Note:*** Cut the tendon as low as possible to have enough length for handling the muscle and attaching it to the tissue chopper disc.

***Note:*** If the TA tendon is not carefully separated from the EDL tendon, both muscles will be dissected together. In this case, the EDL can be removed before processing the TA with the tissue chopper.

8. Wash the TA in a clean Petri dish filled with cold PBS.

Methods video S1. Dissecting the TA, related to step 3

### Formaldehyde fixation

**Timing: 16 h**

The following steps describe formaldehyde fixation to ensure tissue preservation.

9. Transfer the TA into a 15 mL tube with 2 mL of ice cold 4% formaldehyde solution.
10. Place the sample on a rocking platform at 25 rpm, at 4°C, for 16 h.

***Note:*** The steps described here are also suitable for the preparation of samples for immunostaining. For immunostaining, TAs are fixed for 20 min in 2% formaldehyde solution at 4°C.

### Generating thick tissue sections

**Timing: 1 h 30 min**

The following steps describe how to obtain optimal thick tissue sections using a McIlwain tissue chopper (Methods video S2).

**Figure 2:**
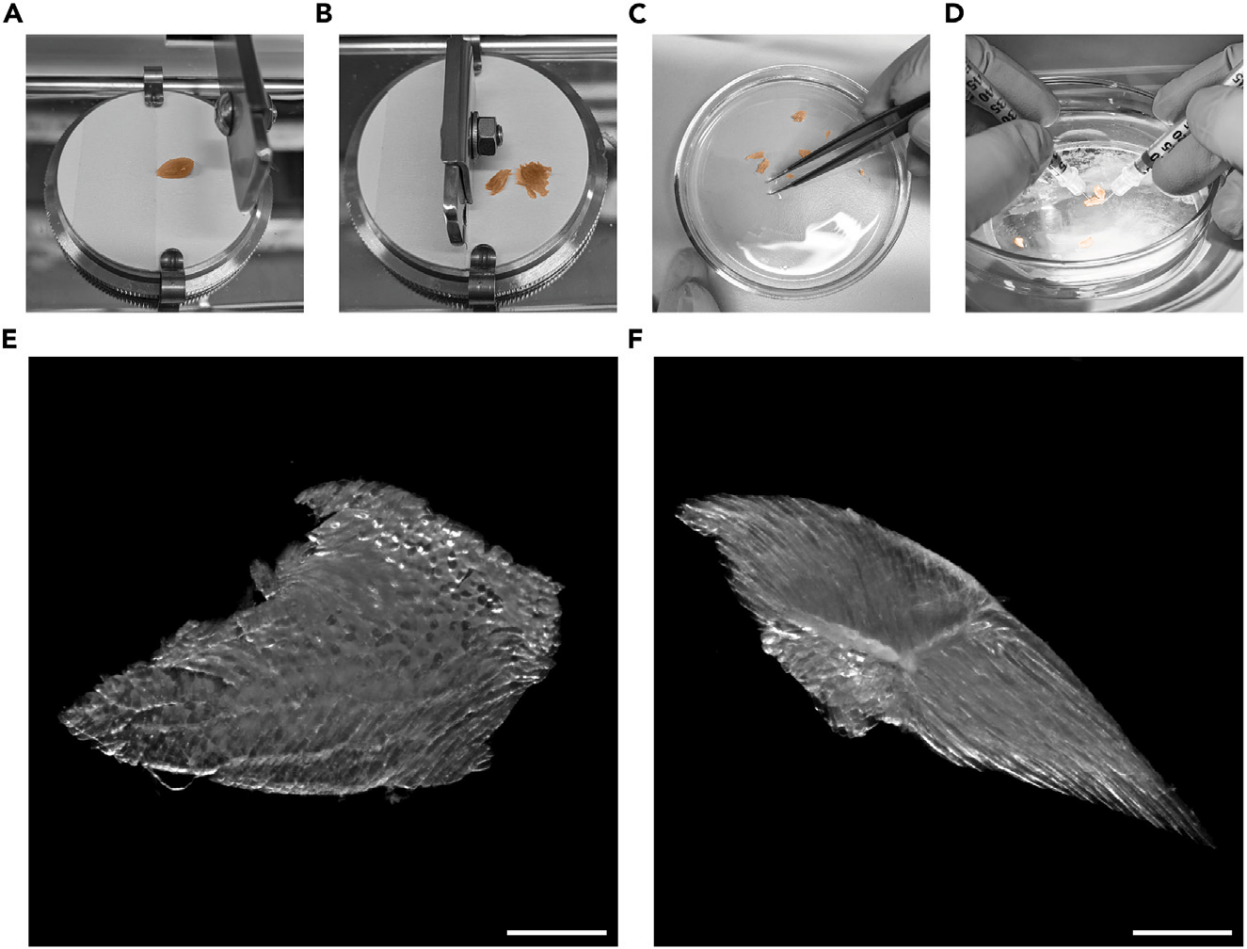
Sectioning the TA using the tissue chopper (A) TA is attached onto the tissue chopper disc, (B) then cut. (C) Sections are transferred to a Petri dish filled with PBS and (D) separated under a stereomicroscope. (E) Examples of a good section with cross sectional appearance and (F) a suboptimal section where fibers were cut longitudinally. Scale bar 1 mm.

11. Wash TA muscle 3 times, for 10 min, with PBS at 4°C.
12. Set up the tissue chopper to cut sections of 300 μm thickness.
13. Dry the TA muscle on a paper towel and place it on the tissue chopper disc.
14. Attach the TA muscle onto the tissue chopper disc, using a piece of tape to hold the muscle by the tendon (Figure 2A).
15. Start the tissue chopper to cut the entire muscle (Figure 2B).
16. Transfer sections into a Petri dish filled with PBS (Figure 2C).
17. Separate sections using insulin needles under a stereomicroscope (Figure 2D).
18. Separate optimal sections (with cross-sectional fibers) from suboptimal sections (with longitudinal fibers) (Figures 2E and 2F). See troubleshooting, problem 1.

***Note:*** Suboptimal sections can be used as staining controls or protocol optimization.

***Note:*** If spatial information needs to be preserved, perform sequential cutting to avoid that sections of different areas mix together. Cut the first region of the tissue, pause the tissue chopper, collect and select sections and repeat the procedure for the other regions (e.g. distal, central, and proximal region of the TA muscle).

***Note:*** Cryosections of fixed muscle frozen in isopentane could be used as an alternative, however using thick sections allows screening of a larger tissue portion at once.

19. Place the sections to be stained in separate 2 mL SafeSeal micro tubes with 1 mL of PBS (see step 24) and proceed to step 25.
20. Collect all remaining sections in 1.5 mL screw neck glass vials with 1 mL of PBS.

***Note:*** Be careful not to damage sections with forceps. Alternatively, use Pasteur pipettes or Moria perforated spoon for transferring sections.

21. Dehydrate samples using a series of freshly prepared MetOH/PBS-Tween washes below, each for 5 min at 4°C:
  a. 25% MetOH / 75% PBS-Tween (1 mL: 250 μL MetOH/750 μL PBS-Tween).
  b. 50% MetOH / 50% PBS-Tween (1 mL: 500 μL MetOH/500 μL PBS-Tween).
  c. 75% MetOH / 25% PBS-Tween (1 mL: 750 μL MetOH/250 μL PBS-Tween).
  d. 100% MetOH (1 mL).
  e. 100% MetOH (1 mL).
22. Store sections at −20°C in 100% MetOH until use.

**Pause point:** If necessary, the dehydrated sections can be stored at −20°C for 6–12 months. Before proceeding to step 24, rehydration is needed (see step 23).

Methods video S2. Generating sections using the tissue chopper, related to step 12

### HCR detection and amplification

**Timing: 2 days**

The following steps describe the probe hybridization and amplification process according to the HCR protocol from Molecular Instruments.

23. Rehydrate sections using a series of freshly prepared MetOH/PBS-Tween washes, each for 5 min at 4°C:
  a. 75% MetOH/ 25% PBS-Tween (1 mL: 750 μL MetOH/250 μL PBS-Tween).
  b. 50% MetOH/ 50% PBS-Tween (1 mL: 500 μL MetOH/500 μL PBS-Tween).
  c. 25% MetOH/ 75% PBS-Tween (1 mL: 250 μL MetOH/750 μL PBS-Tween).
  d. 100% PBS-Tween (1 mL).
24. Transfer the required number of sections to single 2 mL SafeSeal micro tubes. For this experiment we have a stained sample and a negative control:

**Table:** 

Samples	Probe set	Hairpin h1 & h2
Stained	*Ttn*	B2-647
Negative CTR	-	B2-647

***Note:*** The following incubation and washing steps are performed using the ThermoMixer, shaking at 300 rpm, at the indicated temperature.

25. Wash sections with PBS-Tween for 10 min at 19°C–22°C.
26. Treat tissues with 10 μg/mL proteinase K solution for 10 min at 19°C–22°C.

***Note:*** The incubation condition indicated above is optimal for the TA muscle. When using different tissues, proteinase K concentration and time should be optimized to that specific tissue.

27. Wash the sections twice with PBS-Tween at 19°C–22°C for 5 min each.
28. Postfix with 4% formaldehyde solution for 15 min at 19°C–22°C.
29. Wash sections three times with PBS-Tween at 19°C–22°C for 5 min each.
30. Incubate tissues in 150 μL of pre-warmed probe hybridization buffer for 5 min at 37°C.
31. Remove the hybridization buffer and pre-hybridize with 150 μL of pre-warmed probe hybridization buffer for 30 min at 37°C.
32. In the meantime, prepare the probe solution by adding 2.4 μL (16 nM) of the *Ttn* probe set to 150 μL of probe hybridization buffer and prewarm at 37°C, until step 31 is completed.
33. Remove hybridization buffer and
  a. Add 150 μL of the probe solution to the stained sample.
  b. Refresh hybridization buffer for the negative control.
34. Incubate for 16 h at 37°C.

***Note:*** Pre-heat probe wash buffer to 37°C and equilibrate amplification buffer to 19°C–22°C before use.

35. Wash the sections four times for 15 min with 250 μL of probe wash buffer at 37°C.
36. Wash samples twice for 5 min with 5× SSCT at 19°C–22°C.
37. Incubate tissues in 150 μL of equilibrated amplification buffer for 5 min at 19°C–22°C.
38. Remove the buffer and pre-amplify with 150 μL of amplification buffer for 30 min at 19°C–22°C.

***Note:*** During this incubation step, prepare the hairpin solution as indicated in the next step.

39. For each sample, prepare one PCR tube with 3 μL of hairpin h1, and one tube with 3 μL of hairpin h2 (e.g., for the stained sample and the negative control, in separate tubes aliquot 6 μL of hairpin h1 B2-647 and 6 μL of hairpin h2 B2-647).
  a. In a thermocycler, heat hairpins at 95°C for 90 s.
  b. Take out tubes and cool to 19°C–22°C in the dark for 30 min.
40. Mix hairpin h1 and h2 and add them to 150 μL of amplification buffer at 19°C–22°C.
41. Remove the amplification buffer from the sample and add 150 μL of the hairpin solution.
42. Incubate for 16 h in the dark at 19°C–22°C.
43. The next day, remove excess hairpins by using a series of 500 μL 5× SSCT washes at 19°C–22°C.
  a. 2 × 5 min.
  b. 2 × 30 min.
  c. 1 × 5 min.

### Immunofluorescence staining

**Timing: 2 days**

The following steps describe blocking to reduce background and primary and secondary antibody staining.

44. Block sections in PBS-MT for 1 h, on a rocking platform at 25 rpm, in the dark, at 4°C.
45. Perform primary antibody staining:
  a. Incubate the stained sample with primary antibodies and DAPI (see table) by preparing the primary solution in 500 μL of PBS-MT.
  b. Incubate the negative control with DAPI only.

**Table:** 

Primary solution	Isotype	Dilution	500 μL (500 μL/sample)
Laminin	Rat IgG1	1:200	2.5 μL
GFP	Rabbit IgG	1:200	2.5 μL
DAPI	–	1:1000	0.5 μL

46. Incubate samples at 4°C, for 16 h, on a rocking platform at 25 rpm, in the dark.
47. The next day, wash the sections three times using 1 mL of PBS-MT for 1 h at 4°C on a rocking platform at 25 rpm, in the dark.
48. Incubate the stained sample and the negative controls with the secondary solution (see table) in 500 μL of PBS-MT, at 4°C, for 16 h, on a rocking platform at 25 rpm, in the dark.

**Table:** 

Secondary solution	Fluorophore	Dilution	1 mL (500 μL/sample)
Goat anti-Rat IgG	Alexa Fluor 594	1:500	2 μL
Goat anti-Rabbit IgG	Alexa Fluor 488	1:500	2 μL
DAPI	405	1:1000	1 μL

***Note:*** For convenience, incubation of the primary or secondary solution can be performed over the weekend.

49. The next day, wash the samples three times in 1 mL of PBS-MT for 1 h at 4°C on a rocking platform at 25 rpm, in the dark.

***Note:*** While washing the sections, bring Ethyl Cinnamate (ECi) to 19°C–22°C, as ECi is required for the next step.

### Tissue clearing

**Timing: 3 h**

The following steps describe tissue dehydration and clearing to allow imaging of thick sections.

50. Wash the sections three times in PBS-Triton for 20 min at 4°C on a rocking platform at 25 rpm, in the dark.
51. Transfer the sections to 1.5 mL screw neck glass vials and dehydrate the sections in a series of freshly made EtOH solutions for 10 min, each at 19°C–22°C, in the dark:
  a. 25% EtOH (1 mL: 250 μL EtOH/750 μL ddH_2_O).
  b. 50% EtOH (1 mL: 500 μL EtOH/500 μL ddH_2_O).
  c. 75% EtOH (1 mL: 750 μL EtOH/250 μL ddH_2_O).
  d. 100% EtOH (1 mL).
52. After the last dehydration step, incubate the sections once more in fresh 100% EtOH for 30 min at 19°C–22°C, in the dark.

**CRITICAL:** It is important that the sections are fully dehydrated before proceeding to step 53. If samples are not completely dehydrated, the ECi solution will emulsify.

53. Clear the sections by incubating the samples in 100% ECi for at least 30 min at 19°C–22°C, in the dark.

***Note:*** Incubating the sections in ECi over the weekend ensures optimal tissue clearing.

**CRITICAL:** When treating the samples with ECi use airtight 1.5 mL screw neck glass vials, as the ECi may otherwise evaporate. Do not use plastic tubes, since ECi gradually dissolves plastic.

**CRITICAL:** Imaging should be performed within 2 weeks, as the staining intensity decreases significantly after this period. See troubleshooting, problem 4.

**Pause point:** Samples can be stored in the clearing medium at 19°C–22°C in the dark for up to 2 weeks and should only be mounted before imaging.

### Mounting and imaging

**Timing: 3 h**

The following steps describe the procedure to mount the samples and set up the confocal microscope for imaging. Here we perform imaging using a Leica Sp8 confocal microscope, with a 63× water immersion lens (Leica microscope objective HCX PL APO 63×/1.20 W CORR). However, depending on the instruments available in the lab, imaging can be performed using other confocal systems.

54. Superpose two silicon adapters on a glass slide to make a well with a thickness of about 200–300 μm.
55. Place each section into a well with 8 μL of ECi and seal the well with a glass coverslip.

***Note:*** Avoid sample drying by mounting no more than two samples at a time, as imaging takes long and ECi evaporates quickly.

***Note:*** If ECi leaks out of the well or evaporates while imaging, there could be loss of signal. If this happens, carefully remove the glass coverslip and mount the sample in a fresh well with new ECi.

56. Start up the system and open the Leica LAS X software.
57. Locate and focus the sample through the eyepieces by visualizing the nuclei (DAPI channel) using a 63× water immersion lens.
58. In the software, go to configuration, open the laser configuration window, and activate diode 405 and WLL (power 50%).
59. Go to acquisition and configure the beam path using the dye assistant.
  a. Add the fluorochromes and select detectors:
    i. DAPI (PMT).
    ii. Alexa Fluor 488 (PMT).
    iii. Alexa Fluor 594 (HyD).
    iv. Alexa Fluor 647 (HyD).
  b. Choose scanning option (line sequential, 4 sequences).
60. Go to live and for each channel adjust gain and laser power.

**Table:** 

Channel	Fluorophore	Detector	Spectral position (nm)	Laser power (%)	Gain (V)
1	DAPI	PMT1	359–457	10	700
2	Alexa Fluor 488	HyD2	499–520	25	100
3	Alexa Fluor 594	PMT3	590–618	10	750
4	Alexa Fluor 647	HyD4	650–671	25	100

61. Select bidirectional acquisition, image format 1024 × 1024 pixels, scan speed 400 Hz, pinhole 1 AU.
62. Set up a Z-stack of 150–200 μm with a step size of 1 μm.
63. After imaging, save images and Z-stacks and process them using Fiji (Methods video S3).

***Note:*** Following the imaging session, samples can be stored into glass tubes for up to 2 weeks from the completion of this protocol.

Methods video S3. Z-stack of murine TA, related to step 63 The 100 μm Z-stack demonstrates a different antibody and probe penetration. While the laminin signal (magenta) appears mainly at the surface, GFP^+^ satellite cells (marked by white arrows) are visible across most of the stack, and the *Ttn* probe signal is present throughout.

## Expected outcomes

The protocol described here was adapted for staining thick sections of the tibialis anterior (TA) muscle, based on protocols originally designed for whole-mount embryo staining.^3^^,^^4^ Here we integrated an *in situ* Hybridization Chain Reaction (HCR) and immunofluorescence staining to visualize gene expression within defined muscle populations. While not shown here, *in situ* HCR can be multiplexed^5^ by selecting HCR probe sets and amplifiers compatible with immunofluorescence staining, and avoiding spectral overlap.

**Figure 3:**
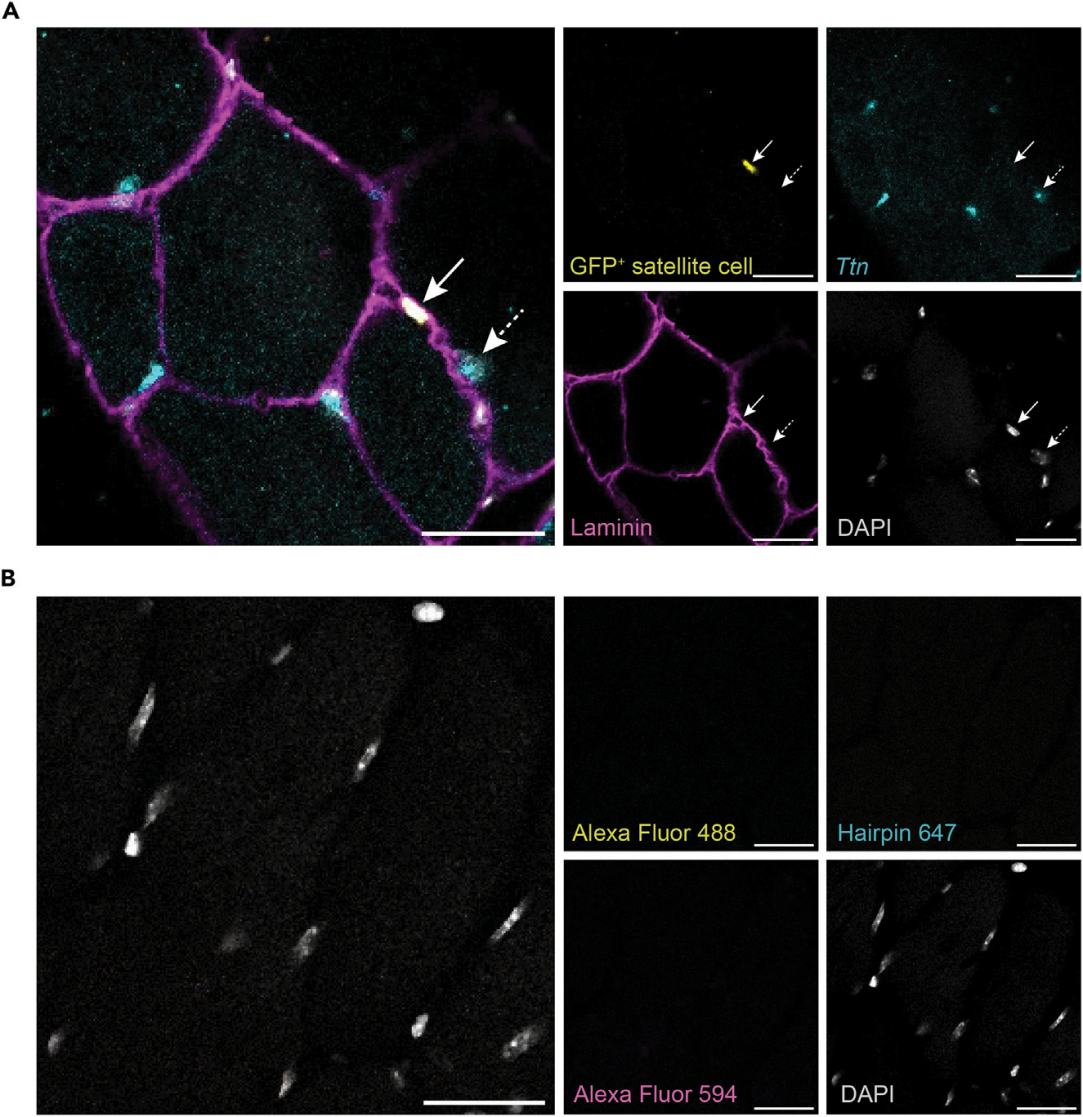
*Ttn* transcript expression in TA muscle sections (A) *Ttn* is expressed in myonuclei (dashed arrow) and myofibers, but not in Pax7-nGFP^+^ satellite cells (solid arrow). (B) Negative control stained with secondary antibodies, B2 amplifier (hairpin h1 and h2), and DAPI. Scale bar 20 μm.

As a proof of concept, we stained thick TA muscle sections with a probe set to detect *Ttn*, a transcript broadly expressed in skeletal muscle. We confirmed that *Ttn* is expressed in myonuclei and myofibers, but not in satellite cells. These populations were easily distinguished by labeling all nuclei with DAPI, the basal lamina surrounding each fiber with an antibody against laminin, and satellite cells with a GFP antibody which recognizes the Pax7-nGFP transgene expressed in the reporter mouse line (Figures 3A and 3B).

**Figure 4:**
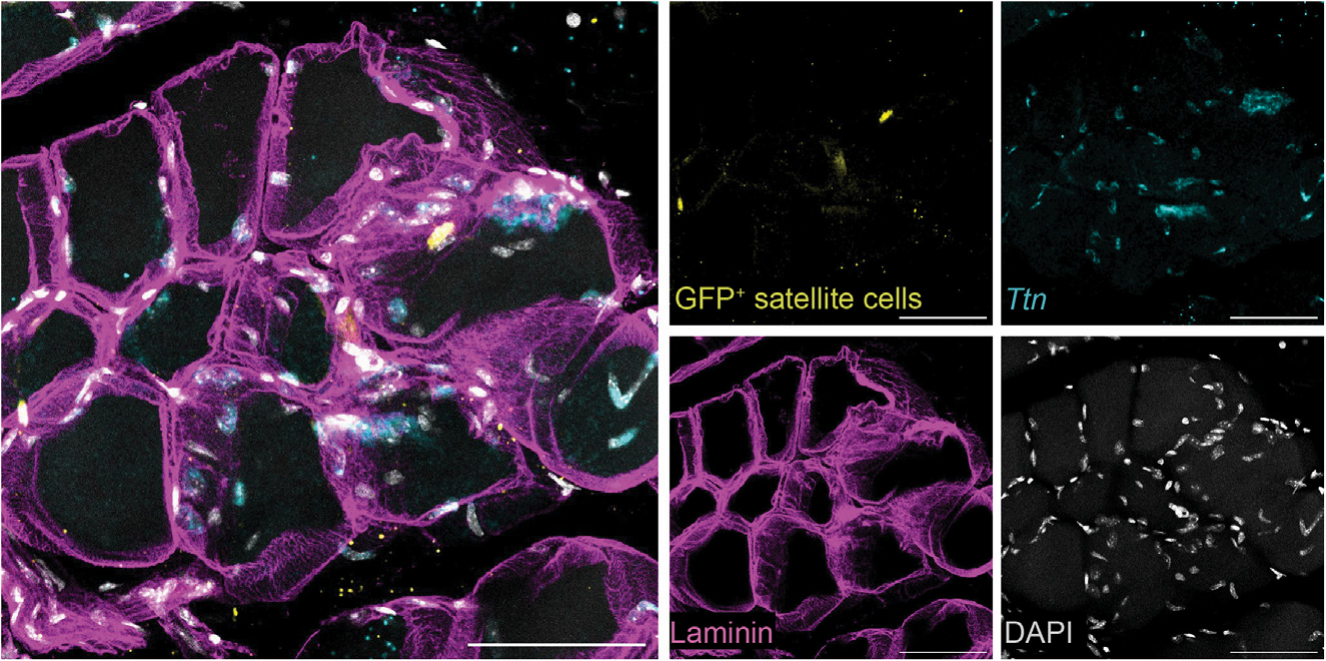
Visualization of rare satellite cells in TA muscle sections Maximum projection of a 20 μm Z-stack made with 1 μm steps. Large Z-stacks allow to easily screen for the presence of rare satellite cells (Pax7-nGFP^+^). Scale bar 50 μm.

Satellite cells are a rare cell population of skeletal muscle stem cells, accounting for about 2–5% of the total nuclei in skeletal muscle and thus their imaging can be challenging. Using thick tissue sections resolves the need of screening numerous 8–10 μm sections, and also facilitates the visualization of these cells within their 3D tissue context (Figure 4, Methods video S3).

**Figure 5:**
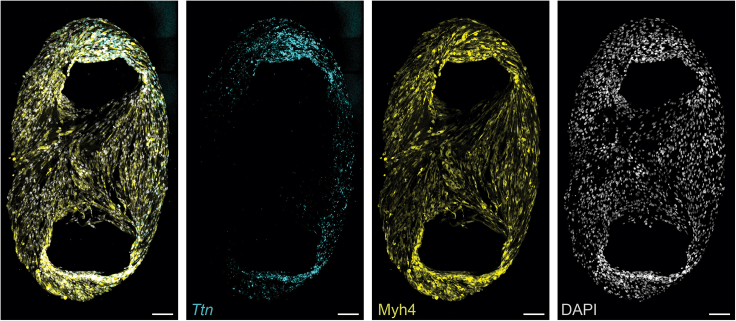
Expression of *Ttn* and Myh4 in differentiated murine myoblasts Maximum projection of a 20 μm Z-stack of TEMs. The expression of *Ttn* transcripts (cyan) and Myh4 (yellow) confirms differentiation of 3D *in vitro* TEMs. Scale bar 100 μm.

To allow deep imaging of thick sections, clearing reagents are necessary. While BABB is commonly used for this purpose, its toxicity poses concerns. The compatibility of other clearing methods with the HCR protocol had not been previously established. Here we demonstrate that Ethyl Cinnamate,^6^ a non-toxic tissue clearing alternative, can effectively substitute BABB, without compromising tissue integrity or imaging quality (Figures 4 and 5, Methods video S3).

To demonstrate the versatility of this protocol, we generated 3D *in vitro* TEMs from primary murine myoblasts. We encapsulated primary myoblasts, 50.000 cells per tissue, in a hydrogel mixture consisting of Collagen I and Matrigel in a 2:1 ratio. We cast the cells into an engineered platform previously used to form engineered heart tissues^7^ in which cells in hydrogel form elastic band-like structures around two elastic pillars in a mold (Figure 5). After 30 min of incubation at 37°C, we cultured the TEMs in Ham-F10 with 20% fetal bovine serum. After 24 h, the medium was replaced by differentiation medium consisting of DMEM and 2% horse serum. TEMs were differentiated for 4 days before fixation and staining, according to the above protocol. In contrast to the thick muscle sections, 3D *in vitro* TEMs are significantly smaller and have reduced thickness (20–30 μm), allowing imaging without requiring tissue clearing. Staining showed the expression of *Ttn* transcripts and Myh4, confirming that 3D *in vitro* TEMs have differentiated and formed myotubes (Figure 5).

## Limitations

Several steps of the HCR protocol can interfere with antibody staining. Although formaldehyde fixation and methanol permeabilization are required to preserve tissue structure, maintain RNA integrity, and facilitate probe penetration, these treatments may not be compatible with certain antibodies. Additionally, the proteinase K treatment alters protein integrity and can affect the quality of antibody staining.

For instance, in a 100 μm Z-stack, the laminin staining appeared more superficial than the GFP staining, indicating limited penetration of antibodies into the tissue section. Instead, the *Ttn* probe signal remained detectable throughout the entire 100 μm stack (Methods video S3).

While not shown here, we provide a list of other antibodies compatible with this protocol, with corresponding working dilutions.

**Table:** 

Antibody	Source	Identifier	Dilution
Anti-Laminin antibody produced in rabbit	Sigma Aldrich	L9393	1:250
Biotin Rat Anti-Mouse CD31	BD Pharmingen	553371	1:500
Anti-GFP antibody	Abcam	ab13970	1:500

## Troubleshooting

### Problem 1

Sections are longitudinal after the muscle is sectioned with the tissue chopper (Related to generating thick tissue sections).

### Potential solution

Examine the sections under a stereomicroscope and separate the suboptimal sections (with longitudinal fibers), from the most optimal (Figures 2E and 2F). Suboptimal sections may be used to titrate antibodies and probes, or to perform control staining.

### Problem 2

No or low signal following HCR (Related to HCR detection and amplification).

### Potential solution

• RNA may be degraded: use RNase-free reagents and consumables, treat tools and surfaces with RNase ZAP.
• Proteinase K incubation time and concentration may be insufficient: test the optimal conditions.
• Verify that correct probe sets and amplifiers were used.
• As probes may not be validated and to exclude issues with the probe design request support from Molecular Instruments.
• Test the probe set concentration (titrate concentrations ranging between 4 to 16 nM).
• Perform imaging within 2 weeks after completion of the protocol.

### Problem 3

High background signal following HCR (Related to HCR detection and amplification).

### Potential solution

• Increase washing time and/or steps.
• Reduce concentration of the probe set.

### Problem 4

**Figure 6:**
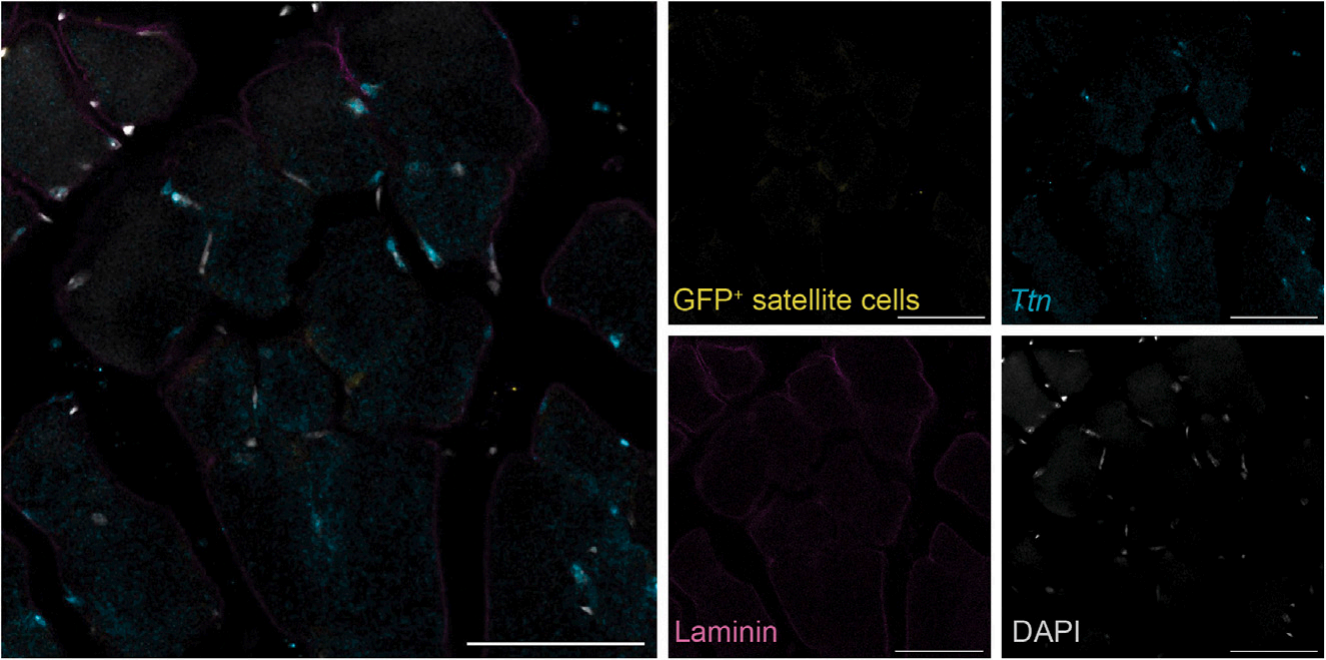
Antibody signal loss While the probe signal is still detectable, antibody signal quickly diminishes following the completion of the protocol (see GFP and Laminin panels). Images were acquired three weeks after staining. Scale bar 100 μm.

No- or low antibody signal following immunostaining (Related to immunofluorescence staining). See staining of Pax7-nGFP and laminin (Figure 6).

### Potential solution

• Increase primary antibody concentration.
• Verify that compatible primary and secondary antibodies were used.
• Reduce the blocking time.
• Lower the formaldehyde solution concentration and/or incubation time.
• Perform imaging within 2 weeks after completion of the protocol.

### Problem 5

High background following immunostaining (Related to immunofluorescence staining).

### Potential solution

• Titrate primary antibody concentration, as the used concentration may be too high.
• Increase the blocking time.
• Increase washing time and/or steps.
• Include a secondary only control to exclude non-specific binding.

## Resource availability

### Lead contact

Further information and requests for resources and reagents should be directed to the lead contact, Fanny Sage (f.g.sage@lumc.nl).

### Technical contact

Technical questions on executing this protocol should be directed to and will be answered by the technical contact, Paola Pisterzi (p.pisterzi@lumc.nl).

### Materials availability

This study did not generate new unique reagents.

### Data and code availability

This study did not generate datasets.

## Acknowledgments

We thank the LUMC animal facility and the LUMC Light and Electron Microscopy facility for providing services and support. We thank S. Tajbakhsh (Institute Pasteur, Paris, France) for providing Tg:Pax7-nGFP mice. This work was supported by the 10.13039/501100009708Novo Nordisk Foundation Center for Stem Cell Medicine (reNEW, grant number NFF21CC0073729) and the Netherlands Organ-on-Chip Initiative, which is an 10.13039/501100003246NWO Gravitation project (024.003.001) funded by the 10.13039/501100003245Ministry of Education, Culture and Science of the government of the Netherlands. Parts of the images were generated with BioRender.

## Author contributions

Conceptualization P.P., F.S., and N.G.; methodology, P.P., C.M.M., O.D., and F.S.; investigation, P.P., C.M.M., O.D., I.d.P., S.B.P., and M.D.; writing – original draft, P.P., C.M.M., and O.D.; writing – review and editing, M.M., C.M., F.S., and N.G.; visualization, P.P., C.M.M., O.D., I.d.P., and S.B.P.; supervision, F.S. and N.G.; funding acquisition, M.M., C.M., and N.G.

## Declaration of interests

The authors declare no competing interests.
